# Supplementary material for: Communications Is Time for Care: An Italian Monocentric Survey on Human Papillomavirus (HPV) Risk Information as Part of Cervical Cancer Screening
Source: J Pers Med. 2022 Aug 26;12(9):1387. doi: 10.3390/jpm12091387 (PMC9505682; doi:10.3390/jpm12091387)
Supplement: Supplementary file 1 [file jpm-12-01387-s001.zip › jpm-1846977-supplementary.pdf]

CERVICAL-CARCINOMA DIAGNOSIS AND ONCOLOGICAL PREVENTION CENTER
QUESTIONNAIRE FOR PATIENTS WITH HPV INFECTION

(The information you provide will be used anonymously)

1) Are you aware of what HPV is?	☐ YES	☐ NO
2) Are you aware of the usefulness of the PAP-TEST?	☐ YES	☐ NO
3) Do you know what COLPOSCOPY consists of?	☐ YES	☐ NO
4) Do you think you have received sufficient and comprehensive information on HPV?	☐ YES	☐ NO
5) Did you search for information about HPV on sources such as INTERNET, SOCIAL MEDIA, TELEVISION?	☐ YES	☐ NO
IF THE ANSWER IS YES		
6) Did you find this information useful?	☐ YES	☐ NO
7) Do you often use this tool to search for medical information?	☐ YES	☐ NO
8) Did you research this information ON INTERNET before consulting your doctor?	☐ YES	☐ NO
9) Do you think that the Internet use may have changed the relationship with the doctor?	☐ YES	☐ NO
10) In particular, do you think it may have improved your emotional state?	☐ YES	☐ NO
11) Do you think it appropriate to integrate the information found on internet with your doctor?	☐ YES	☐ NO
12) Do you feel more informed when you find information on the internet?	☐ YES	☐ NO
13) On the contrary, do you think this information contributed to the anxiety in her?	☐ YES	☐ NO
14) Have you ever found confusing and / or contradictory information?	☐ YES	☐ NO
15) Is the word High or Low Risk HPV in the report a concern for you?	☐ YES	☐ NO
16) Does your doctor give you full explanations?	☐ YES	☐ NO
17) Do you think there is a need for further information tools?	☐ YES	☐ NO
18) Have you ever needed psychological support?	☐ YES	☐ NO
19) Do you think it could be useful and would you benefit from a psychological support service?	☐ YES	☐ NO
20) What would you suggest to improve communication and information on HPV?		

Supplementary Figure S1: questionnaire offered to women during cervical cancer screening consisting of 20 questions of which only one is open-ended; the remaining 19 can only be answered yes or no.
